# Cardiac perfusion imaging using hyperpolarized ^13^c urea using flow sensitizing gradients

**DOI:** 10.1002/mrm.25713

**Published:** 2015-05-20

**Authors:** Angus Z. Lau, Jack J. Miller, Matthew D. Robson, Damian J. Tyler

**Affiliations:** ^1^ Division of Cardiovascular MedicineRadcliffe Department of Medicine, University of OxfordUnited Kingdom; ^2^ Department of PhysiologyAnatomy and Genetics, University of OxfordUnited Kingdom; ^3^ Department of PhysicsClarendon Laboratory, University of OxfordUnited Kingdom

**Keywords:** hyperpolarization, urea, first‐pass perfusion, cardiac, ^13^C

## Abstract

**Purpose:**

To demonstrate the feasibility of imaging the first passage of a bolus of hyperpolarized ^13^C urea through the rodent heart using flow‐sensitizing gradients to reduce signal from the blood pool.

**Methods:**

A flow‐sensitizing bipolar gradient was optimized to reduce the bright signal within the cardiac chambers, enabling improved contrast of the agent within the tissue capillary bed. The gradient was incorporated into a dynamic golden angle spiral ^13^C imaging sequence. Healthy rats were scanned during rest (n = 3) and under adenosine stress‐induced hyperemia (n = 3).

**Results:**

A two‐fold increase in myocardial perfusion relative to rest was detected during adenosine stress‐induced hyperemia, consistent with a myocardial perfusion reserve of two in rodents.

**Conclusion:**

The new pulse sequence was used to obtain dynamic images of the first passage of hyperpolarized ^13^C urea in the rodent heart, without contamination from bright signal within the neighboring cardiac lumen. This probe of myocardial perfusion is expected to enable new hyperpolarized ^13^C studies in which the cardiac metabolism/perfusion mismatch can be identified. Magn Reson Med, 2015. © 2015 The Authors. Magnetic Resonance in Medicine published by Wiley Periodicals, Inc. on behalf of International Society for Magnetic Resonance in Medicine. This is an open access article under the terms of the Creative Commons Attribution License, which permits use, distribution and reproduction in any medium, provided the original work is properly cited. **Magn Reson Med 75:1474–1483, 2016. © 2015 The Authors. Magnetic Resonance in Medicine published by Wiley Periodicals, Inc. on behalf of International Society for Magnetic Resonance.**

## INTRODUCTION

In the treatment of patients with coronary artery disease, the main clinical question is the identification of viable hibernating myocardium and discrimination from nonviable necrotic tissue 1. Hibernating myocardium is characterized by a metabolism‐perfusion mismatch with intact metabolism and impaired contractility due to reduced myocardial perfusion 2. Restoration of blood flow to viable tissue restores normal function and improves clinical outcome, but results in unnecessary cost and clinical risk for patients with nonviable tissue 3. Noninvasive imaging methods to assess both metabolism and perfusion in the ischemic heart are necessary to guide the clinical decision to proceed with revascularization.

Preclinical small animal models of heart disease are widely used and enable the study of both structural and functional remodeling following myocardial infarction when imaged using cardiac magnetic resonance imaging. Quantitative evaluation of myocardial blood flow using MRI can be achieved by dynamic imaging of the first passage of a Gd‐DTPA bolus, in both rodent 4, 5, 6, 7, 8 and human hearts 9, 10, 11. Noncontrast arterial spin labeling (ASL) methods have also been used to noninvasively measure perfusion in rodents 12, 13, 14, 15, 16, 17, 18, 19, with potential for quantification in humans 20, 21, although scan times remain long and are difficult to integrate into a larger preclinical MR study of cardiac function.

The dynamic nuclear polarization and dissolution method produces a liquid injectable agent that is hyperpolarized, providing signal increases in a ^13^C‐labeled molecule relative to thermally polarized spins in excess of 10,000‐fold 22. This new magnetic resonance based metabolic imaging modality has the potential to assess in vivo metabolic changes in real‐time. In the most common application of this technique, infusion of ^13^C‐labeled pyruvate, and subsequent imaging allows assessment of key biochemical pathways, including anaerobic metabolism by observation of ^13^C‐labeled lactate as well as flux into the TCA cycle 23, 24, 25. The infusion of a hyperpolarized agent has also long been proposed as a method for perfusion measurement due to the relatively long signal lifetime and low background signal of the images 26, 27, 28. The linear signal response of the agent with respect to concentration also enables direct quantification and the ability to detect balanced ischemia in the organ of interest. The initial proof‐of‐concept studies were performed using ^13^C‐labeled urea 22, an endogenous molecule, and subsequently both ^13^C urea 29 and the nonendogenous blood pool agent, HP001 30 have been used as probes of perfusion within the kidneys, in models of cancer, and in large animal cardiac models 31. The tracer ^13^C t‐butanol has also been used as a perfusion probe in the brain 27 and in tumor models 32, and is notable due to its freely diffusible nature, which can simplify modeling of tissue perfusion.

Despite this, application to the rodent heart has remained challenging due to the high spatial and temporal resolution required to resolve the first‐pass of the agent into the myocardium. In particular, typical cardiac wall thicknesses of 2 mm in conjunction with high signal within the lumen leads to high spatial resolution requirements to visualize the agent within the tissue capillary bed without contamination from the neighboring blood pool. Intracoronary infusion by coronary catheterization allows direct visualization of the hyperpolarized signal within the capillary bed 31, but is highly invasive and is typically not feasible for small animal imaging.

In this work, we have investigated the feasibility of imaging the first‐pass of a bolus of hyperpolarized ^13^C urea through the rat heart. We have used flow‐sensitizing bipolar gradients 33, 34 to reduce the bright signal within the cardiac chambers, enabling improved contrast of the agent within the tissue capillary bed of the myocardium. The new method is demonstrated in the healthy in vivo rat heart during rest and stress conditions, and is expected to enable new hyperpolarized ^13^C studies in which the cardiac metabolism/perfusion mismatch can be identified.

## METHODS

All experiments were performed on an Agilent 7 Tesla (T) MRI system (Agilent, Santa Clara, CA). All animal investigations conformed to Home Office Guidance on the Operation of the Animals (Scientific Procedures) Act (HMSO) of 1986, to institutional guidelines, and were approved by the University of Oxford Animal Ethics Review Committee.

### Pulse Sequence

**Figure 1:**
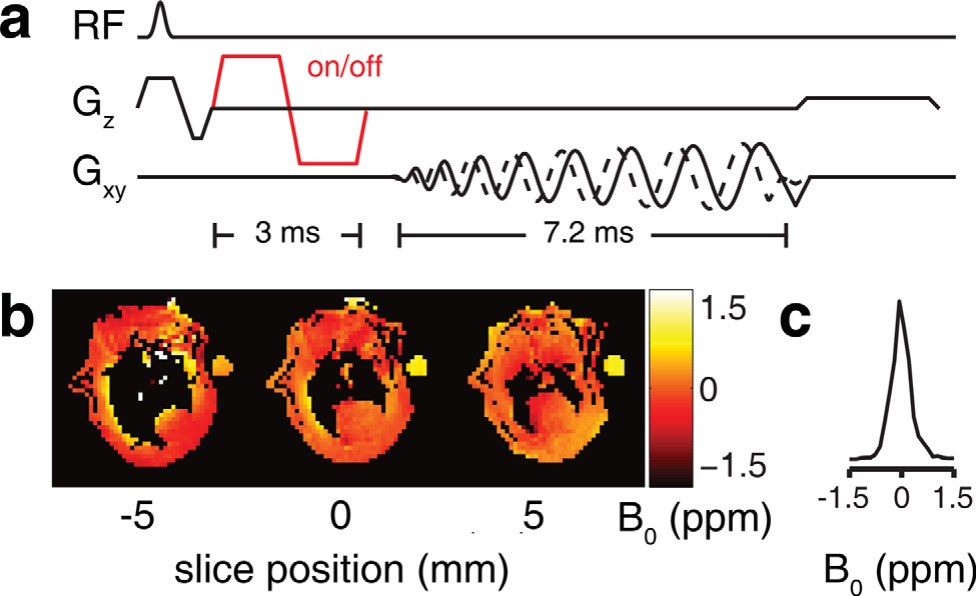
Flow sensitized ECG‐gated spiral sequence for first‐pass perfusion imaging of ^13^C in the heart. **a**: The bipolar flow sensitizing gradient (red) is toggled in alternate TRs, dephasing signal from within the cardiac chambers. **b,c**: Separately acquired ^1^H B_0_ maps are used to calculate the expected linewidth (∼0.5 ppm) in the slice of interest. A thermally polarized urea frequency reference is also visible by the side of the animal.

Figure 1a shows the flow sensitive ECG‐gated, golden‐angle spiral sequence used to obtain axial dynamic ^13^C urea images in the heart. Each interleave was obtained by rotating the previous interleave by the golden angle $\left(3-\sqrt{5}\right)180{}^{\circ}\approx 137.508{}^{\circ}$; this results in near uniform coverage of k‐space with adaptive temporal resolution when combining a variable number of interleaves 35. The readout duration (7.24 ms for ^13^C) was chosen to minimize off‐resonance blurring artifacts, based on a proton B_0_ field map acquired in a 1 cm slab encompassing the target slice of interest (Figures 1b and 1c). The expected linewidth in vivo was typically 0.5 ppm, or 35 Hz for ^13^C at 7T, ensuring that the readout was short enough to avoid image blurring.

Flow contrast was incorporated by inserting a bipolar gradient after the end of the slice‐selective excitation and before the start of the readout. Flowing spins accumulated phase proportional to their velocity in the presence of the bipolar gradient, and the mechanism of signal suppression was by intravoxel dephasing of spins with different velocities. The chosen direction of the bipolar gradient was along the long axis of the heart to optimize the amount of dephasing of flowing spins. The required bipolar gradient amplitude was calibrated by obtaining ECG‐gated ^1^H images with the same spatial resolution. The gradient amplitude was chosen by maximizing signal suppression of rapidly flowing spins within the lumen of the heart, while minimizing signal loss from the myocardium and the blood within this tissue. The required gradient amplitude was then scaled by the ratio γ_H_/γ_C_ to account for reduced phase accrual for ^13^C relative to ^1^H.

### Phantom Study

The performance of the flow spoiling effect was investigated using a flow phantom experiment. A PVC tube (inner diameter 6 mm) was placed through the magnet bore, through which tap water was driven under steady flow at varying volumetric rates (0, 2, 4, 8, 12, 16 mL/s). The maximum Reynolds number for these flow rates was 1700, corresponding to laminar flow. Flow encoded transverse ^1^H FLASH images were acquired (echo time [TE] = 5 ms, repetition time [TR] = 15 ms, FA 7°, field of view [FOV] 12 × 12 mm^2^, matrix 48 × 48, acquired in‐plane resolution 0.25 × 0.25 mm^2^, slice thickness 5 mm). The bipolar gradient was oriented along the direction of flow, the gradient duration was set to 3 ms, and the gradient amplitude was varied (0, 5, 40, 80 mT/m). The first gradient moments ( $m_{1}=\int G\left(t\right)tdt$) corresponding to these values were 0, 11, 87, and 168 mT·ms^2^/m. The velocities (VENC = π/(γm_1_)) corresponding to 180° phase accrual during the bipolar gradient were 105, 13.5, and 7.0 cm/s.

To demonstrate the effect of the bipolar gradient direction on the flow spoiling effect, the flow rate was fixed to 4 mL/s, and a second set of images were obtained with the flow direction across the image. The bipolar gradient was oriented either parallel or perpendicular to the direction of flow, and the gradient amplitude was varied from 0 to 80 mT/m (first gradient moments: 0 to 168 mT·ms^2^/m). A velocity map describing flow within the tube in both image orientations was computed by phase contrast between velocity encoded image pairs with VENC = 105 cm/s. The images were filtered in k‐space to a final resolution of 2.1 × 2.1 mm^2^ to achieve similar spatial resolution to the in vivo experiments, described below.

### Animal Handling

Male Wistar rats (n = 6, weight = 503 ± 44 g) were scanned in this study. Anesthesia was induced at 2.5–3% isoflurane in oxygen and nitrous oxide (4:1, total of 2 L/min). Anesthesia was maintained by means of 2% isoflurane delivered to, and scavenged from, a nose cone during the experiment. A tail vein catheter was placed for intravenous infusion of hyperpolarized ^13^C urea. A separate tail vein catheter was placed in the contralateral tail vein for delivery of adenosine during the stress‐induced hyperemia experiments, described below. Animals were then placed in a home‐built animal handling system 36. Body temperature was maintained using air heating, and a two‐lead ECG for cardiac gating was recorded using leads placed subcutaneously into the upper forelimbs.

### Stress Perfusion

Coronary vasodilation was induced by continuous adenosine infusion (concentration 3 mg/mL, flow rate 280 μg/kg/min) into the tail vein using a small animal infusion pump (Harvard Apparatus, MA). The volume of the injection line was filled with adenosine, and hyperpolarized ^13^C urea was administered 10 min after the start of infusion through a separate tail vein catheter. Adenosine infusion was maintained throughout the ^13^C scan. No adverse effects were detected during or following adenosine infusion.

### Hyperpolarization

A stock ^13^C urea solution (6.4 M) with OX063 trityl radical (15 mM) was prepared in a glycerol/water solution (6:4 ratio by weight) 37. Fifty microliter samples of the stock solution were mixed with 3.5 μL of Gd‐DOTA (final sample concentration 2.3 mM) (Dotarem, Guerbet, France). Polarization was performed using a prototype DNP hyperpolarizer at 93.957 GHz and 100 mW for 120 min. Dissolution was performed at a pressure of 10 bar and a temperature of ∼170 °C in 5 mL of 0.38 mM EDTA in water, resulting in a final injected substrate concentration of 64 mM. Due to the short T_1_ of ^13^C urea in very low field (∼0.2 mT adjacent to the polarizer), the hyperpolarized solution was transferred from the polarizer into a low‐field (2–10 mT) magnetic holder within 1 s. The holder consists of a plastic bottle with four permanent neodymium magnets attached outside, in a quadrupolar arrangement. The solution was then transferred to the fringe field (2 mT) of the magnet before injection. The liquid state polarization level was 12% at the end of injection, as assessed by a separate thermal equilibrium phantom experiment. Two mL of prepolarized ^13^C urea was injected over 20 s by means of the tail vein.

### MRI Acquisitions

^1^H images were acquired using a 72‐mm inner diameter dual‐tuned birdcage transmit/receive ^1^H/^13^C coil. ^13^C images were acquired using the same volumetric coil for RF transmission, and a two‐channel surface receive array (two 2 × 4 cm^2^ elements oriented in the left–right direction) for signal reception (Rapid Biomedical GMBH, Rimpar, Germany). A volume covering a 70‐mm slab including the heart was used for shimming using a 3D gradient echo automated shim routine 38. A prospectively triggered segmented cine FLASH sequence was used to assess cardiac function in a transverse mid‐ventricular slice (TE 1.36 ms, TR 4.6 ms, FOV 80 × 80 mm^2^, acquired in‐plane resolution 0.8 × 0.8 mm^2^, slice thickness 5 mm, FA 20°).

As described above, the required bipolar gradient amplitude to null flowing spins in the heart was calibrated using ^1^H images with the same reconstructed spatial resolution as the ^13^C acquisition. The ^1^H calibration scan was compatible with multiple gradient echo readout types, and was demonstrated using both Cartesian (TE 5 ms, TR 1 RR, FA 20°, FOV 80 × 80 mm^2^, matrix 128 × 128, slice thickness 5 mm, 2 averages) and a golden angle spiral trajectory (TE 4.3 ms, TR 1 RR, FA 20°, FOV 80 × 80 mm^2^, 12 shots for full FOV coverage, matrix 128 × 128, acquired in‐plane resolution 0.625 × 0.625 mm^2^, slice thickness 5 mm, 256 total shots, 137.508° rotation between shots, readout duration 3.4 ms). The calibration images were smoothed using a Hamming filter to match the in‐plane resolution of the ^13^C images (2.3 × 2.3 mm^2^). The scan time was the same (40 s) for each acquisition. The data was obtained with multiple averages (Cartesian) or with a large number of interleaves (non‐Cartesian spiral) to reduce respiratory motion artifacts.

Images of the first‐pass of a bolus of hyperpolarized ^13^C urea through the heart were obtained using the ECG‐gated golden angle spiral sequence (TE = 4.4 ms, TR 1 RR, FA 20°, FOV 80 × 80 mm^2^, 4 shots for full FOV coverage, matrix 64 × 64, acquired in‐plane resolution 1.25 × 1.25 mm^2^, slice thickness 5 mm, excitation pulse duration 0.5 ms, readout duration 7.24 ms, bipolar gradient amplitude G_flow_ = 170 mT/m, total duration 3 ms, first gradient moment m_1_ 332 mT·ms^2^/m, maximum encoding velocity (VENC) 14.1 cm/s). The flow sensitivity and VENC were kept constant by taking the bipolar flow gradient amplitude determined from the ^1^H calibration scan and scaling it by approximately four (γ_H_/γ_C_) to account for the reduced phase accrual for ^13^C relative to ^1^H. A single value for the gradient amplitude (170 mT/m) was chosen for all subsequent experiments. The transmitter frequency was set to the frequency of a thermally polarized 6.4 M ^13^C urea phantom placed on the side of the coil, which was shimmed to within 1 ppm of the heart. The scan was started before injection, and images were acquired for at least 1 min after the start of injection, corresponding to at least 400 time points at the typical heart rates in the study.

### Image Reconstruction

The multishot spiral trajectory was predicted using a premeasured gradient impulse response function 39. The non‐Cartesian k‐space samples were gridded using the nonuniform fast Fourier transform 40. The density weighting for k‐space samples was calculated for each set of interleaves 41. To correct for bulk B_0_ shifts in the image, the center frequency was automatically identified by means of autofocus off‐resonance correction by summing all time frames with signal, reconstructing a series of images by demodulating at different frequencies, and selecting the frequency which minimized the imaginary component of the images 42. This frequency was used for subsequent reconstruction of each individual time frame. A sliding window was used to group spiral interleaves (6 interleaves per image). The images were Hamming filtered to a reconstructed in‐plane resolution of 2.3 × 2.3 mm^2^. The receive coil sensitivities were estimated from two individual coil images obtained by summing together all time frames with signal, and the resulting sensitivities were used to combine the channels in each individual time frame 43.

### Data Analysis

The heart was manually segmented using the anatomical cine images into right ventricle (RV), left ventricle (LV), and myocardial tissue. The ^13^C urea magnitude images were used for data analysis. The signal means within each compartment were baseline corrected using the mean LV signal with no agent, and then normalized to the maximum signal within the LV. The image signal‐to‐noise ratio was calculated by dividing the image intensity in each frame by the standard deviation of image intensity in frames before ^13^C urea arrival.

Areas under the curve (AUC) were calculated for each compartment, and an ad hoc measure of myocardial perfusion was taken to be the ratio between tissue AUC and LV AUC. The rationale for this calculation is to remove any polarization or concentration differences between different injections. Additionally, the integration procedure improves the signal‐to‐noise ratio (SNR) in the individual compartment time courses. An unpaired, two‐tailed Student's t‐test was used to compare AUC ratios between rest and stress conditions. Statistical significance was considered at the *P* < 0.05 level. Myocardial perfusion reserve was estimated by taking the ratio between the group perfusion ratios at stress and at rest.

## RESULTS

### Phantom Study

**Figure 2:**
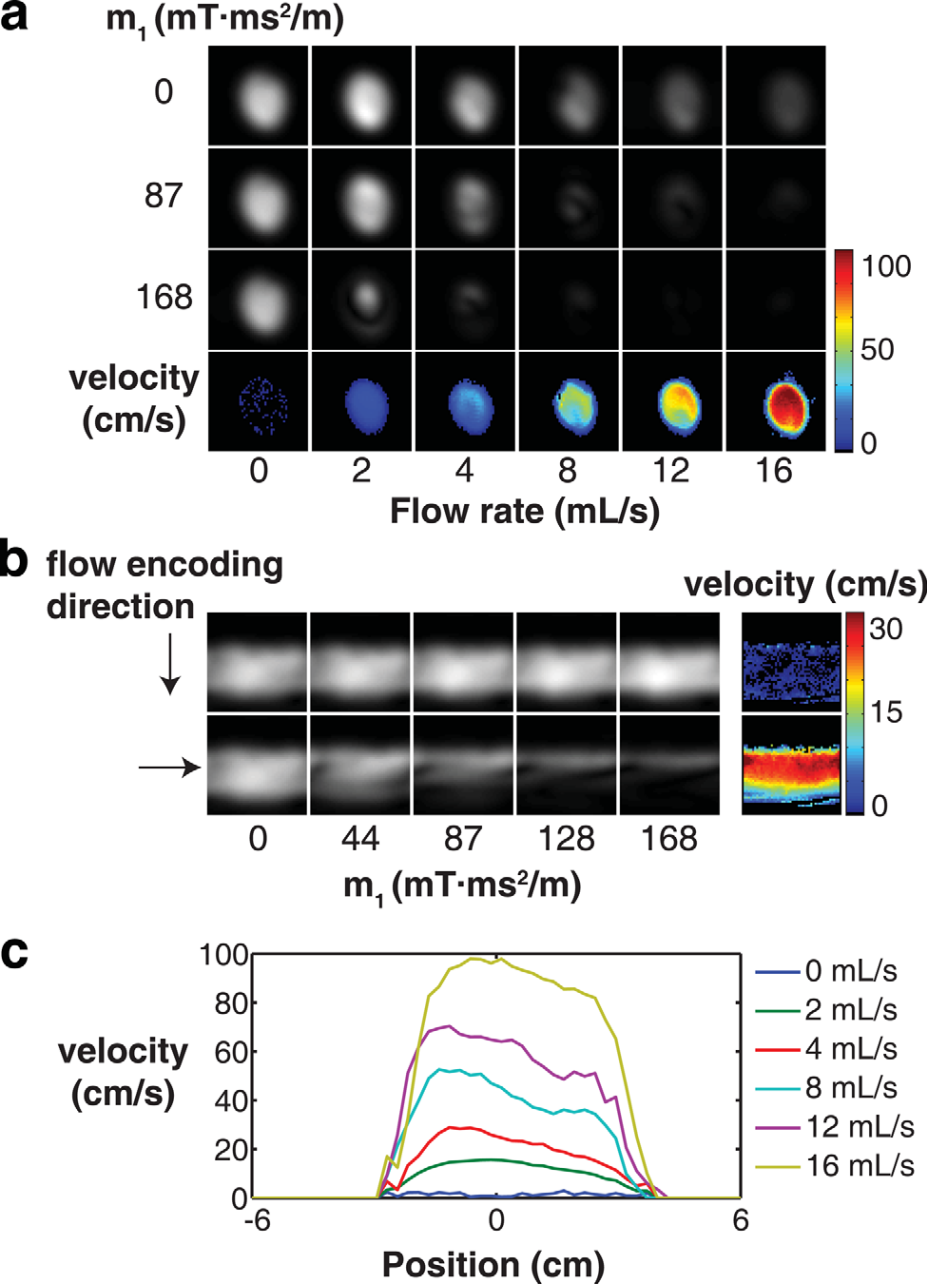
Demonstration of the flow dephasing effect in a flow phantom. **a**: Images transverse to the direction of flow (through‐slice) are obtained with varying flow rate and flow encoding bipolar first gradient moment. The images are normalized to the image intensity in the nonflow encoded, zero flow rate image. The images are cropped to a 12 × 12 mm^2^ FOV. **b**: The flow rate was fixed at 4 mL/s, and images with the direction of flow (in‐plane) are obtained with varying flow encoding gradient direction and first moment. The dephasing effect occurs when the flow sensitizing gradient is parallel to the direction of flow, as shown by the phase contrast velocity map on the right. This is consistent with (**c**) the measured intravoxel velocity distribution at a flow rate of 4 mL/s.

Figure 2 shows flow‐sensitized images in a tube with flowing water. In Figure 2a, images were obtained with varying flow and different bipolar gradient amplitudes. The bipolar gradient direction was oriented parallel to the direction of flow. The signal intensity was spoiled at high flow and high gradient amplitude. At an intermediate flow encoding gradient amplitude, the signal was retained at low flow rates; however, the signal was dephased at higher flow rates. In Figure 2b, the flow rate was fixed at 4 mL/s, and the direction of the bipolar gradient was oriented either perpendicular or parallel to the direction of flow. This demonstrated that the efficiency of spoiling depended on the bipolar gradient direction. At a flow rate of 4 mL/s, the maximum velocity within the tube was 29 cm/s. Spins moving at this velocity accrue an approximate phase of 360° at m_1_ = 87 mT·ms^2^/m, which was consistent with the signal attenuation shown in Figure 2c. For the subsequent in vivo studies, the bipolar gradient was oriented along the long axis of the heart, as this is the predominant direction of blood flow during the cardiac cycle.

### In Vivo Study

**Figure 3:**
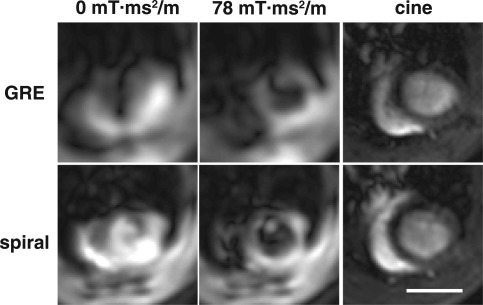
In vivo ^1^H images obtained using the flow sensitizing gradient using either Cartesian gradient echo readout (top row) or golden angle spiral readout (bottom row). The flow sensitizing gradient (m_1_ = 78 mT·ms^2^/m, VENC = 15 cm/s) dephases signal from within the lumen, while stationary anatomy is preserved. A cine image is shown as an anatomical reference. The images are cropped to a 25 × 25 mm^2^ FOV, and the scale bar indicates 10 mm.

Proton calibration scans were performed to determine the required bipolar gradient amplitude necessary to spoil flowing ^13^C signal from within the cardiac chambers. Figure 3 shows the results of the calibration scan using both Cartesian gradient echo and non‐Cartesian spiral readouts. The required gradient amplitude for typical flow rates within the rat heart was determined to be 40 mT/m (m_1_ = 78 mT·ms^2^/m, VENC = 15 cm/s). At this amplitude, the signal from within the lumen was dephased, while signal from stationary spins was preserved. The anatomical cine reference demonstrates that the non–flow‐suppressed signal comes from the tissue.

**Figure 4:**
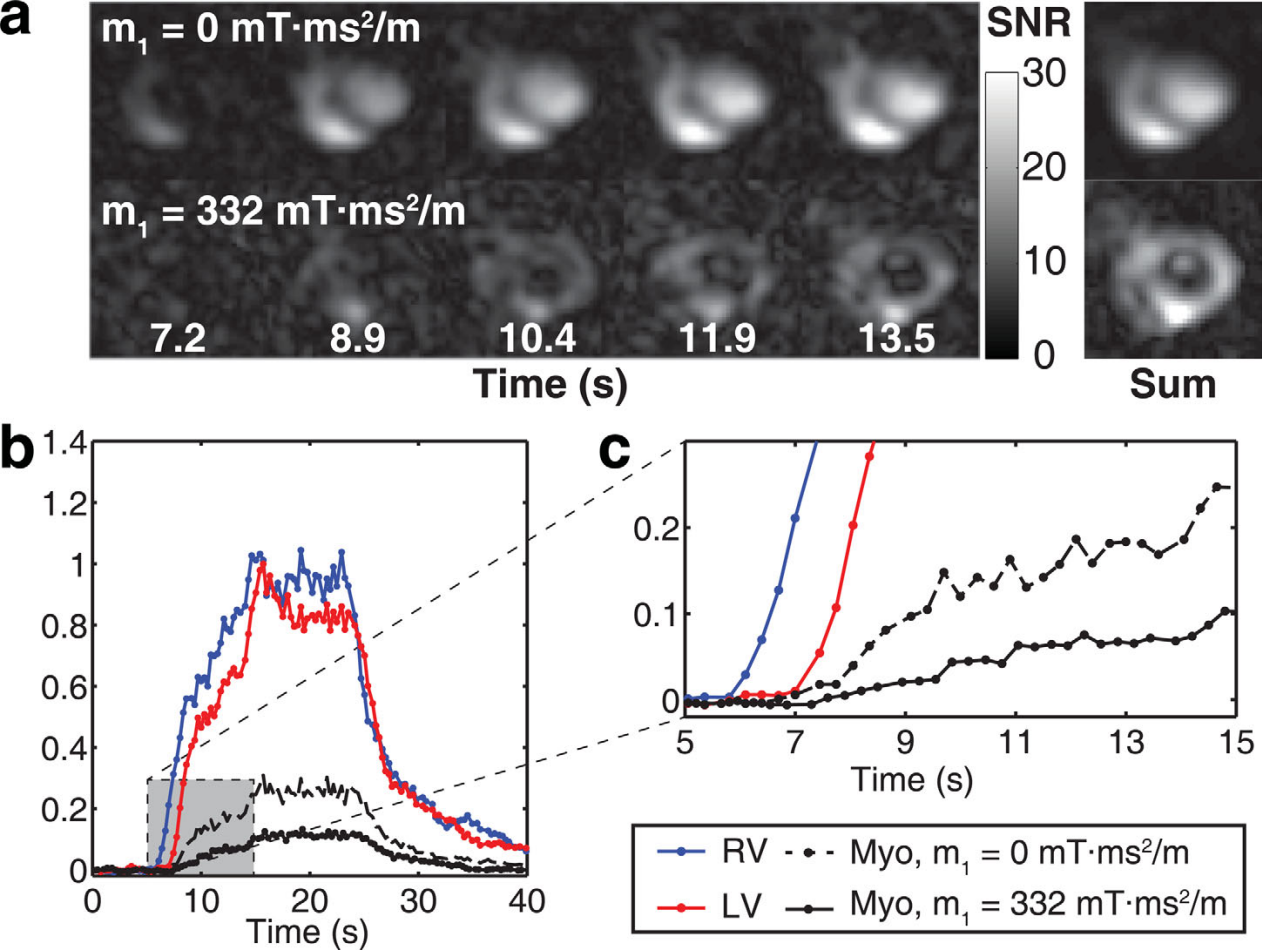
In vivo data showing the first pass of urea in the rat heart. **a**: Images from every 5^th^ frame are shown without (m_1_ = 0 mT·ms^2^/m) and with (m_1_ = 332 mT·ms^2^/m, VENC = 14.1 cm/s) flow sensitivity. The images are cropped to a 25 × 25 mm^2^ FOV. **b**: Time course covering the first pass of the urea signal within the RV, LV, and myocardium. The blood pool signals in the RV (blue) and LV (red) are segmented from the m_1_ = 0 mT·ms^2^/m images, and the myocardial signals are shown with no flow encoding (black, dashed) and with flow sensitization (black, solid). In this image, the maximum image SNR in the LV was 28. **c**: The zoomed portion of the time course shows the different arrival times of the agent within the compartments.

Figure 4 shows representative images of the first passage of hyperpolarized ^13^C urea through the heart. ^13^C urea was visualized in the entire heart, but when the bipolar gradient was turned off, urea within the myocardial wall could not be resolved due to insufficient in‐plane resolution. The maximum image SNR averaged in the left ventricular ROI was found to be 37 ± 12 (n = 6). The flow‐sensitizing bipolar gradient (amplitude 170 mT/m, m_1_ = 332 mT·ms^2^/m, VENC = 14 cm/s) dephased signals with varying velocities within a voxel, improving visualization of the slowly moving ^13^C urea within the cardiac wall. The time courses (Figures 4b and 4c) show the different arrival times of the agent within the RV, LV, and myocardium. In particular, the flow sensitizing gradient reduces contamination between ^13^C urea in slowly moving blood within the myocardial capillary bed and ^13^C urea in flowing blood within the cardiac chambers.

**Figure 5:**
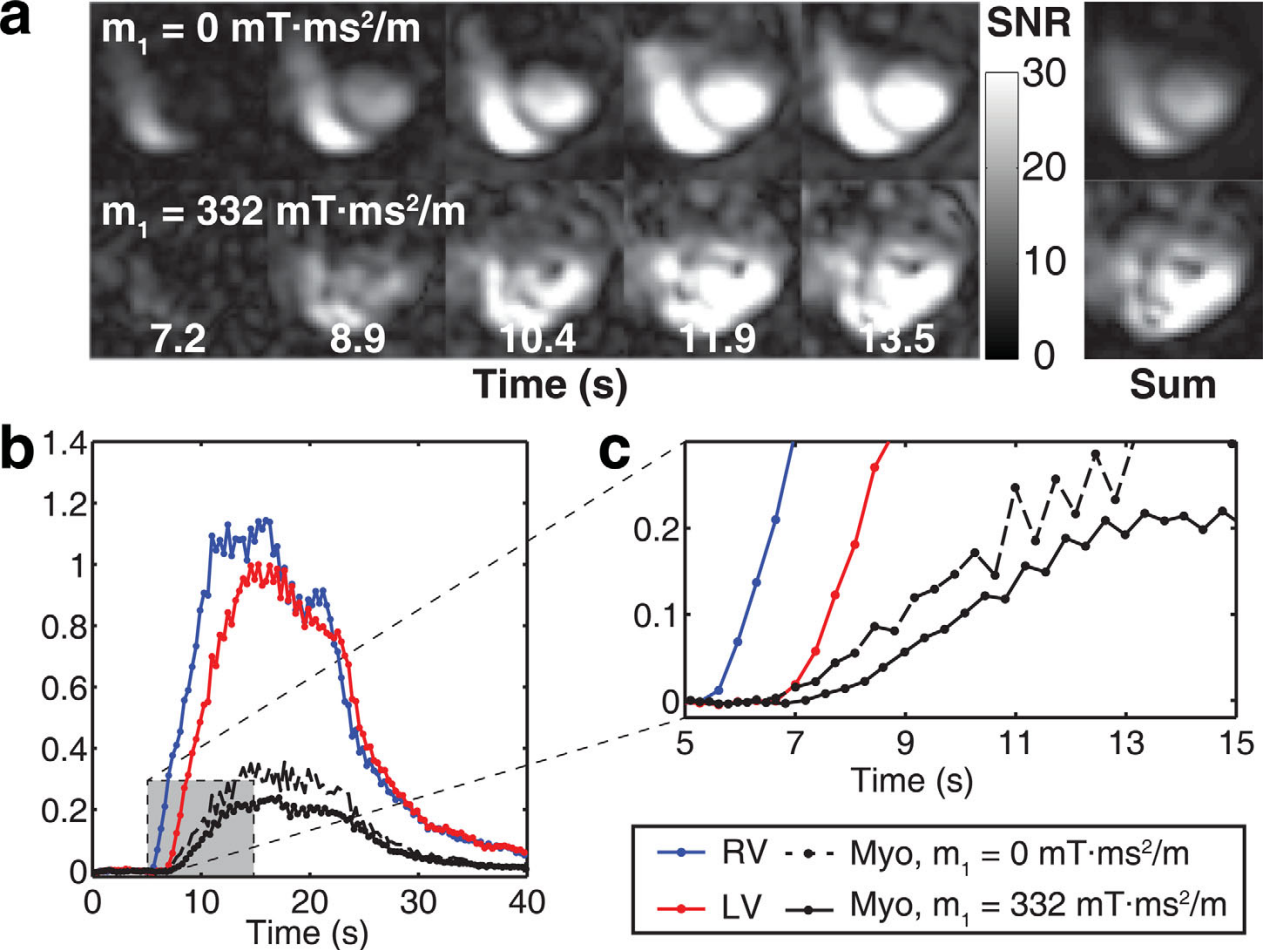
In vivo data showing the first pass of urea in the rat heart during adenosine stress‐induced hyperemia. **a**: Images from every 5^th^ frame are shown without (m_1_ = 0 mT·ms^2^/m) and with (m_1_ = 332 mT·ms^2^/m, VENC = 14.1 cm/s) flow sensitivity. The images are cropped to a 25 × 25 mm^2^ FOV. **b**: Time course covering the first pass of the urea signal within the RV, LV, and myocardium. The blood pool signals in the RV (blue) and LV (red) are taken from the m_1_ = 0 mT·ms^2^/m images, and the myocardial signals are shown with no flow encoding (black, dashed) and with flow sensitization (black, solid). In this image, the maximum image SNR in the LV was 42. **c**: The zoomed portion of the time course shows the different arrival times of the agent within the compartments.

Figure 5 shows the detection of adenosine stress‐induced hyperemia in the rat heart using hyperpolarized ^13^C urea. The mean heart rate did not change significantly during adenosine infusion (rest condition: 382 ± 7 beats per min, stress condition: 392 ± 33 beats per min, *P* = 0.32). The images show increased maximum myocardial ^13^C urea signal relative to images acquired during rest, as well as an increase in the rate of arrival of the agent in the tissue.

**Figure 6:**
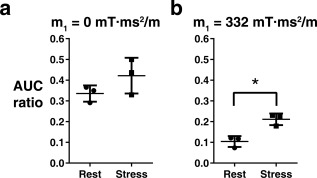
Semi‐quantitative measures of myocardial perfusion using hyperpolarized ^13^C urea. The area under the curve (AUC) ratio between myocardial tissue and left ventricle is shown using the nonflow sensitized (m_1_ = 0 mT·ms^2^/m) (**a**) and flow sensitized (m_1_ = 332 mT·ms^2^/m) (**b**) images. A significantly different ^13^C urea ratio within the tissue between rest and adenosine stress‐induced hyperemia is detected using the flow sensitized images (*P* < 0.05).

Figure 6 shows a statistical comparison between myocardial ^13^C urea signals obtained during rest and during adenosine stress‐induced hyperemia. Myocardial perfusion was evaluated by normalizing myocardial tissue AUC by left ventricular AUC. In Figure 6a, the ratio calculated when the flow‐sensitizing gradient was turned off was not significantly different between rest and stress conditions. However, when the flow‐sensitizing gradient was turned on, as shown in Figure 6b, we observed a significant two‐fold increase in the ratio of myocardial tissue AUC to left ventricular AUC between rest (0.10 ± 0.03) and stress (0.21 ± 0.03) conditions (*P* < 0.05).

## DISCUSSION

### Significance

Measurements of myocardial perfusion in rodents are challenging because of the high spatial and temporal resolution required. The combination of small heart size, rapid heart rate, and limited quiescent periods during the cardiac cycle have thus far restricted use of hyperpolarized agents to perfusion measurements in larger, relatively stationary structures such as the kidneys, cancers, and in larger animal models. In this study, we have demonstrated the feasibility of using flow‐sensitizing bipolar gradients to improve visualization of hyperpolarized ^13^C urea within the rodent myocardium. Hyperpolarized ^13^C urea images were shown to detect an increase in perfusion during low‐flow infusion of adenosine, a coronary vasodilator.

It is anticipated that this development will enable new paradigms of hyperpolarized ^13^C studies in the heart. In particular, the potential to assess both metabolism and perfusion by combining information from infusions of hyperpolarized ^13^C‐pyruvate and ^13^C‐urea will allow a direct visualization of perfusion‐metabolism mismatches in the heart. This will provide a more complete understanding of ischemia‐reperfusion injury and tissue viability following myocardial infarction. Furthermore, hyperpolarized exams are rapid by nature of the short T_1_ of the hyperpolarized ^13^C agent, and can be incorporated into a wider preclinical MR exam of small animal cardiac function with only a marginal increase in study time.

### Physiological Interpretation of Results

Adenosine induces coronary vasodilation without a concomitant increase in heart rate. The two‐fold increase in hyperpolarized ^13^C urea signal following adenosine stress‐induced hyperemia relative to the rest condition is consistent with previous measurements of myocardial perfusion reserve in rats, using first‐pass Gd‐DTPA imaging 5, fluorescent microspheres 18 and contrast‐enhanced ultrasound 44. The myocardial ring is well resolved with the flow‐sensitized contrast, suggesting that regional perfusion deficits following myocardial infarction can be detected using this technique.

### Comparison to Contrast‐Enhanced Perfusion Measurements

Conventional contrast‐enhanced perfusion measurements using Gd‐based contrast agents 4, 5, 6, 7, 8, 9, 10, 11 are mature and simple to implement, so it is of value to consider the advantages of using a hyperpolarized agent to measure perfusion. The main benefits of using hyperpolarized ^13^C urea are its direct signal‐concentration proportionality, in vivo safety, and the short scan time which can be beneficial in the acute setting. Furthermore, while we demonstrate the proof‐of‐principle use of ^13^C urea to monitor perfusion following a single infusion, future studies will investigate the feasibility of using a co‐polarization of ^13^C urea and metabolically active ^13^C pyruvate to provide cardiac perfusion‐metabolism information in a single scan. This would be important in patients with cardiovascular disease and impaired renal function who cannot tolerate a gadolinium chelate infusion.

### Other Hyperpolarized Perfusion Strategies

The main development in this work is the introduction of flow‐sensitizing gradients in the context of hyperpolarized cardiac imaging. Other methods to visualize the myocardium include higher resolution imaging of the hyperpolarized ^13^C bolus by traversing further out in k‐space. This results in a large SNR penalty, and moreover, achieving high spatial resolution typically requires increasing the number of interleaves, degrading the temporal resolution in the final images. It may be possible to use a slice‐selective SSFP sequence in combination with dual‐labeled [^13^C, ^15^N] urea which possesses a long T_2_ 29, but the fidelity of such pulses in the heart and the short TRs required would need to be investigated. In particular, it is not clear that a long slice‐selective echo train 27 with duration on the order of T_2_ would be compatible with imaging of rapidly moving hyperpolarized magnetization, as the RR interval (∼150 ms) is shorter than the reported in vivo T_2_s of 200 ms for ^13^C urea and 5 s for [^13^C, ^15^N] urea. This long pulse sequence would introduce significant blurring over the cardiac cycle, and would also result in a bright blood pool and excessive loss of magnetization as fresh blood enters the slice.

The use of an adiabatic spin‐echo pair (the semi‐LASER sequence) 45 is an attractive imaging strategy for hyperpolarized experiments due to the insensitivity to transmit gain and in‐phase spectra in slice‐selective acquisitions 46. Furthermore, flow‐sensitizing gradients placed around the 180° pulse suppress hyperpolarized signals from flowing spins and enable discrimination between intracellular and extracellular metabolites 37, 47, 48, 49. Typically, the spin‐echo experiment is started after the full injection of ^13^C‐labeled compound is complete, as adiabatic pulses are generally not adiabatic below a certain power level that typically occurs in the fringe field of the transmit coil. This delayed start ensures that the hyperpolarized signal will not be saturated during the injection, but suggests that first‐pass imaging of a prepolarized agent using adiabatic spin‐echo pulses is not straightforward.

### Artifacts and Limitations

As described in the Methods, the spoiling efficiency of the flow‐sensitizing gradient is related to the velocity distribution within the voxel along the direction of the uniaxial bipolar gradient. In this study, the single gradient pulse was oriented parallel to the cardiac long axis, which was chosen as this is the predominant direction of blood flow within the heart. However, this scheme does not suppress flow in either lateral direction, which may become important near the apex of the heart. Nevertheless, we note that the two‐fold increase in hyperpolarized ^13^C urea signal following adenosine stress‐induced hyperemia is made possible by reducing the signal within the cardiac lumen using flow sensitizing gradients; the corresponding nonflow sensitized images do not show this increase in signal. This suggests that in this experimental setup, the degree of blood pool suppression is sufficient to enable detection of large perfusion changes using hyperpolarized ^13^C urea in this experimental setup.

It is interesting to consider whether signal attenuation results from the imaging gradients themselves. The first gradient moment of the spiral trajectory oscillates and reaches a maximum of 316 mT·ms^2^/m at the end of the 7.24 ms readout, similar in magnitude to the bipolar gradient used in this study. Spiral trajectories follow a similar circular trajectory in velocity k‐space, resulting in phase accrual and blurring for in‐plane flow over the duration of the readout 50. It is difficult to assess whether this results in signal attenuation in the ^13^C images in practice as the spiral readout is kept constant for each time frame, but we note the fact that there is bright signal within the lumen in the nonflow encoded images. This is consistent with no flow‐related phase accrual at the center of k‐space, so any low spatial resolution structures (i.e. the left ventricle) will be preserved in the resulting images. Also, the image quality and SNR is similar between the flow‐encoded Cartesian and spiral ^1^H images (Fig. 3), suggesting that any flow attenuation is minimal.

In the flow‐encoded images, bright signal was observed colocalized to the right ventricle as well as within the lumen of the left ventricle. The appearance of signals within the cardiac chambers is presumably due to insufficient dephasing of slowly moving blood within the chambers. It is important to note that there is a spatial resolution dependence on the flow‐sensitizing gradient strength 34, and that different applications may require a different set of parameters to properly null the signal within the contaminating blood pool.

It may be possible to improve the flow sensitization by using more bipolar gradient directions to suppress a general intravoxel velocity distribution, which would come at the expense of temporal resolution. Alternatively, the amplitude of the bipolar gradients could be reduced to introduce a velocity‐encoded phase change between ^13^C images. Segmentation of the images based on velocity would then separate flowing (lumen) and stationary (myocardial) signals.

The hyperpolarized ^13^C urea solution was infused over a period of 20 s, which is longer than the circulation time (< 10 s) in a rat. Thus, there is likely recirculation of the signal even before the end of the infusion. This is a limitation of the experimental protocol, and the long infusion was chosen to maximize the number of frames during which ^13^C urea was present in the heart. We note that the initial upslope of the hyperpolarized signal into the heart is different between rest and stress conditions (Figures 4 and 5). This is a phenomenon of the first passage of a contrast agent. Moreover, the observation of this difference is enabled by using the bipolar flow sensitizing gradient, indicating that we are indeed measuring aspects of the first pass of urea into the myocardium. Urea is nontoxic and can be safely tolerated at higher doses and shorter infusion times than those used in this study 29, 51, and future studies will optimize the bolus timing to improve the first pass nature of a perfusion exam.

### Alternative Methods for Reducing the Blood Pool Signal

Flow‐sensitizing bipolar gradients were used in this study to reduce signal contribution of ^13^C urea in the blood pool, by intravoxel dephasing of flowing spins with different velocities. In this section, we discuss other methods to reduce the blood pool signal, which include spatially selective saturation of inflowing spins 52 as well as separation of signal within the blood pool by comparison to an input function 53.

Saturation of hyperpolarized magnetization using spectral‐spatial RF pulses has previously been used to remove signal from hyperpolarized metabolic products (i.e. lactate) which flow into the imaged slice of interest 52. Saturation of hyperpolarized magnetization within the imaged slice of interest and subsequent imaging of a bolus of inflowing magnetization has previously been described for renal perfusion imaging 54. This approach requires careful placement of the imaging slice to avoid saturation of the upstream arterial signal. In particular, it would be challenging to use this technique when the arterial or luminal signal is also within the slice of interest, as is the case in the heart.

Signal modeling of the blood pool signal using a single‐tissue compartment model can be used to estimate the fractional blood volume in each voxel, by comparison to an input function. A model of this type has previously been used to describe the signal behavior of hyperpolarized agents within brain, liver, and tumor 32. It may then be possible to fit the myocardial signal to a single‐compartment model, but it is important to note that each voxel in the heart contains significant partial volumes of myocardium, slowly flowing myocardial blood within the capillaries, as well as rapidly moving blood within the lumen. To aid the fitting, an arterial input function may be extracted from the signal within the cardiac lumen, and a fractional partial volume between tissue and cardiac lumen may be obtained from the anatomical cine images. Nevertheless, further investigation is required to determine feasibility of this approach.

### Sequence Improvements

The short TR per spiral interleave (∼11 ms) and typical heart rates in rats (150 ms per cardiac cycle) suggests that acquisition of multiple slices per cardiac cycles is feasible, which will provide full volumetric assessment of perfusion in the rodent. As the single 5 mm slice used in this study covered approximately half of the myocardium, thinner slices will permit evaluation of localized disease in basal, mid‐ventricular and apical locations.

### Quantitative Measurements of Absolute Perfusion

In this study, myocardial perfusion was quantified by taking the ratio between areas under the signal time curve in the myocardial tissue and in the left ventricle. Normalization by the left ventricular input is intended to remove differences in ^13^C polarization, urea concentration, and transmit/receive sensitivities between experiments. A comparison between this ratio at rest and during adenosine stress‐induced hyperemia was then used to obtain an estimate of myocardial perfusion reserve.

Absolute perfusion values would enable a quantitative comparison of perfusion between states where a global change in blood flow occurs. The ability to distinguish between normal perfusion and a balanced ischemic state would improve the sensitivity of the technique to disease. In principle, calculating a blood flow rate (in units of mL/min/g tissue) should be possible using a hyperpolarized agent, because there is no signal background and there is a linear relationship between signal intensity and concentration. The main challenge in obtaining a quantitative estimate of perfusion is correctly accounting for the polarization decay over time, similar to the short half‐life (∼2 min) in H_2_^15^O‐PET perfusion measurements 55.

Quantitative models typically rely on characterization of the relationship between the arterial input function and the tissue signal. Estimation of the tissue impulse response function can be performed using model‐based (Fermi) constrained deconvolution or by model‐free singular value decomposition, similar to previous quantitative perfusion measurements using ^13^C agents in kidneys, cancer, and brain 28, 30.

## CONCLUSIONS

The feasibility of a new flow sensitized method for imaging the first passage of a bolus of hyperpolarized ^13^C urea through the rodent heart was demonstrated and quantitatively evaluated. A flow‐sensitizing bipolar gradient was optimized to dephase flowing spins within the lumen of the heart, and was incorporated into a spiral imaging pulse sequence. A two‐fold increase in myocardial perfusion relative to rest was detected during adenosine stress‐induced hyperemia, consistent with a myocardial perfusion reserve of two in rodents. This probe of myocardial perfusion is anticipated to enable new applications in hyperpolarized ^13^C MRI.
